# The glycoprotein hormone receptor (LGR1) influences Malpighian tubule secretion rate in *Rhodnius prolixus*

**DOI:** 10.1242/jeb.249357

**Published:** 2024-12-10

**Authors:** Areej N. Al-Dailami, Angela B. Lange, Ian Orchard

**Affiliations:** Department of Biology, University of Toronto Mississauga, Mississauga, ON, Canada, L5L 1C6

**Keywords:** Triatomine, Diuretic hormones, Serotonin, Feeding, Diuresis

## Abstract

In the hemipteran *Rhodnius prolixus*, successful post-prandial diuresis is accomplished through the synergistic actions of the peptidergic diuretic hormone RhoprCRF/DH and the biogenic amine 5-hydroxytryptamine (5-HT), and by an antidiuretic hormone RhoprCAPA-2 that terminates diuresis by inhibiting this synergy. Lateral neurosecretory cells (NSCs) in the mesothoracic ganglionic mass release RhoprCRF/DH, while midline NSCs release RhoprCAPA-2 during blood feeding. These NSCs co-express GPA2/GPB5, a conserved glycoprotein hormone involved in various physiological processes across bilaterians. This study investigated the influence of GPA2/GPB5 signaling on Malpighian tubule (MT) fluid secretion in *R. prolixus*. GPB5-like immunoreactivity in lateral and midline NSCs decreased following a blood meal, suggesting release and a role in diuresis. Downregulating the GPA2/GPB5 receptor LGR1 via RNA interference resulted in an increased basal fluid secretion rate in MTs, which was inhibited by the antidiuretic hormone RhoprCAPA-2. dsLGR1 treatment reduced the effects of RhoprCRF/DH and 5-HT on MT secretion and eliminated their synergism. RT-qPCR revealed that the expression of the diuretic and antidiuretic hormone receptors decreased in MTs of dsLGR1-injected insects, indicating that GPA2/GPB5 influences the expression of these other receptors. Downregulating LGR1 resulted in a smaller blood meal size and disrupted the normal time course of diuresis. As LGR1 is the most abundantly expressed G protein-coupled receptor gene in *R. prolixus* MTs, our results suggest that GPA2/GPB5 signaling has a critical role in regulating the timing and success of water retention in the unfed state, and in the complex processes associated with feeding and diuresis in *R. prolixus*.

## Teaser

**Summary:** Glycoprotein hormone GPA2/GPB5 signaling is crucial for regulating diuresis in *Rhodnius prolixus*, influencing fluid secretion and the interplay of diuretic and antidiuretic hormones during blood feeding.

## INTRODUCTION

The kissing bug *Rhodnius prolixus*, a major vector of Chagas disease, is an obligate blood feeder that can consume many times its unfed body mass in a single short feed. A post-prandial diuresis is then initiated that can eliminate up to 50% of the volume of the blood meal within 3 h, thereby lowering the insect's mass and concentrating the nutrients from the meal (Orchard et al., 2021). In *R. prolixus*, as with other insects, the Malpighian tubules (MTs) play a critical role in diuresis, and as they are not innervated, their fine control comes under the influence of the neuroendocrine system that releases amines and neuropeptides as diuretic or antidiuretic hormones (see Orchard and Lange, 2020). These hormones act upon the MTs via a variety of G protein-coupled receptors (GPCRs) linked to second messenger systems that influence V-ATPase, ion transporters and aquaporins (Stanisçuaski et al., 2013; Esquivel et al., 2016; Kolosov and O'Donnell, 2019; Orchard and Lange, 2020; Orchard et al., 2021; Dow et al., 2021).

Much has been discovered about the control of diuresis in *R. prolixus*, and other model insects, but the post-genomic era has brought new insights by identifying novel diuretic and antidiuretic hormone-signaling pathways whilst validating existing models (see Kolosov and O'Donnell, 2019; Dow and Davies, 2006; Dow et al., 2021; Orchard et al., 2021). Thus, in *R. prolixus*, the transcripts for receptors of the previously identified diuretic hormones serotonin (5-hydroxytryptamine, 5-HT) and *R. prolixus* corticotropin-releasing factor-like diuretic hormone (RhoprCRF/DH) have been identified, as have the transcripts for the receptor of the *R. prolixus* anti-diuretic hormone (RhoprCAPA-2) (Paluzzi et al., 2010, 2015; Lee et al., 2016; Orchard et al., 2021). Neurosecretory cells (NSCs) expressing 5-HT, CRF/DH or CAPA, are located in the mesothoracic ganglionic mass (MTGM) and release their products at feeding from neurohemal sites found on the abdominal nerves (Lange et al., 1989; Te Brugge et al., 2002a, 2011; Paluzzi and Orchard, 2006; Orchard, 2006; Paluzzi et al., 2008). 5-HT and RhoprCRF/DH act synergistically to stimulate maximum secretion rate by *R. prolixus* MTs, and RhoprCAPA-2 acts as an antidiuretic hormone by inhibiting the effects of 5-HT and eliminating the synergy between 5-HT and RhoprCRF/DH (Paluzzi et al., 2012).

As alluded to earlier, transcriptomes and microarrays provide molecular insights into the functional aspects of the MTs, allowing for the development of hypothetical models for their receptors and second messengers, and ion and water transport mechanisms (Dow and Davies, 2006; Overend et al., 2015; Esquivel et al., 2016; Kolosov and O'Donnell, 2019; Orchard and Lange, 2020; Dow et al., 2021; Orchard et al., 2021; Agard et al., 2024; Halberg and Denholm, 2024). The strength of these studies lies in unanticipated discoveries and one such discovery was recently reported for *R. prolixus* (Orchard et al., 2021). Thus, the most abundant GPCR transcript in the MT transcriptome is that of the leucine-rich repeat-containing G protein-coupled receptor 1 (LGR1) for the glycoprotein hormone GPA2/GPB5, which is approximately 6-fold more abundant than the transcript for Rhopr5HTR2b and 60-fold more abundant than the transcript for Rhopr-CRF/DH-R2B (Orchard et al., 2021). This discovery is all the more interesting for the fact that the lateral NSCs expressing RhoprCRF/DH in the MTGM also co-localize with GPB5-like immunoreactivity, and the midline NSCs expressing CAPA also co-localize with GPB5-like immunoreactivity (Al-Dailami et al., 2023, 2024). Thus, there is the intriguing possibility that the glycoprotein hormone GPA2/GPB5 may be released along with the diuretic hormone RhoprCRF/DH immediately at feeding, but also along with the anti-diuretic hormone RhoprCAPA-2, which is released to inhibit diuresis some hours later. Thus, GPA2/GPB5 might also have actions along with RhoprCRF/DH and RhoprCAPA-2 on the MTs. This paper explores these possibilities.

## MATERIALS AND METHODS

### Animals

All experiments were performed on fifth instar *Rhodnius prolixus* Stål 1859. The *R. prolixus* colony is maintained at 28°C and 50% humidity in an incubator in the dark at the University of Toronto Mississauga, as outlined by Orchard et al. (2021). Briefly, insects are fed through an artificial feeding membrane on defibrinated rabbit blood (Cedarlane Laboratories Inc., Burlington, ON, Canada) once in each instar. Both male and female fifth instars (4–5 weeks post-feeding as fourth instars) were used in this study. All insects used in this work had a similar feeding and body mass history.

### Chemicals

Serotonin hydrochloride (5HT, Sigma-Aldrich, Oakville, ON, Canada) was freshly prepared as a 1 mmol l^−1^ stock solution in deionized water. RhoprCAPA-2 and RhoprCRF/DH were custom synthesized by GenScript (Piscataway, NJ, USA) at >95% purity, prepared as 1 mmol l^−1^ stocks in deionized water and stored in aliquots at −20°C. Both peptides and 5-HT were diluted in *R. prolixus* physiological saline (150 mmol l^−1^ NaCl, 8.6 mmol l^−1^ KCl, 2 mmol l^−1^ CaCl_2_, 4 mmol l^−1^ NaHCO_3_, 34 mmol l^−1^ glucose, 8.5 mmol l^−1^ MgCl_2_, 5 mmol l^−1^ Hepes, pH 7) to the appropriate concentrations.

### Whole-mount immunohistochemistry

Central nervous systems (CNSs) from unfed and fed fifth instars were dissected in *R. prolixus* physiological saline at room temperature. Fixation and staining with a primary anti-GPB5 antiserum was performed as described in Al-Dailami et al. (2022). The anti-GPB5 antiserum was used at 1:1000 in phosphate buffered saline (PBS, 6.6 mmol l^−1^ Na_2_HPO_4_/KH_2_PO_4_, 150 mmol l^−1^ NaCl, pH 7.4) with 0.4% Triton-X, 2% normal goat serum (NGS) and 2% bovine serum albumin (BSA). The anti-GPB5 antiserum was custom made against a conserved sequence within GPB5 (CDSNEISDWRFP) (Rocco and Paluzzi, 2020) and kindly provided by Prof. J. P. Paluzzi, York University, Canada. The secondary antiserum was 1:600 Alexa 488-conjugated goat anti-rabbit antibody (Life Technologies, Carlsbad, CA, USA). Preabsorbed GPB5 antiserum using the antigen showed no GPB5-like staining in CNS samples, as demonstrated previously (Al-Dailami et al., 2022), confirming the specificity of the antisera. Images were acquired, processed and prepared as previously described using a confocal microscope (LSM-800, Carl Zeiss, Jena, Germany) (Al-Dailami et al., 2022). Intensity staining of GPB5-like NSCs from pre- and post-fed *R. prolixus* was quantified using ImageJ software (https://imagej.net/). Each group of lateral NSCs in a MTGM was traced and staining intensity measured and averaged (*n*=5–6 MTGMs). All six mid-line NSCs in a MTGM were traced and staining intensity measured and averaged (*n*=6–7 MTGMs). Grayscale values ranging from 0 (minimum intensity) to 255 (maximum intensity) were assigned to each image. All images were treated consistently with respect to scale and number of slices in the *Z*-stacks.

### Reverse transcription quantitative PCR (RT-qPCR) analysis

MTs from fifth instar *R. prolixus* were used in this study. Total RNA from the various portions of the MTs was extracted, and cDNA was synthesized as previously described (Al-Dailami et al., 2022). Quantitative gene expression was analyzed by the 2^−ΔCT^ method (Schmittgen and Livak, 2008), using the geometric average expression of two reference genes, *rp49* (60S ribosomal protein) and *β-actin*. The output values obtained using 2^−ΔCt^ method were calculated relative to 1000 copies of the reference genes. To check knockdown after dsRNA injection, transcript levels were quantified and relative gene expression shown as fold-change relative to the gene expression of the control samples, using the 2^−ΔΔCT^ method following the geometric mean of the *β-actin* and *rp49* reference genes (Schmittgen and Livak, 2001). Each experiment had 4–5 biological replicates, each containing 2 technical replicates. The sequences of the primers used for amplification are shown in Table S1.

### Double-stranded RNA synthesis and delivery

Two non-overlapping fragments of LGR1 were prepared by PCR by conjugating the T7 RNA polymerase promoter (5′-taatacgactcactatagggaga-3′) to the 5′ end of the gene-specific primers (Table S1), as previously described (Al-Dailami et al., 2022). As a control, dsRNA based on the ampicillin resistance gene (dsARG) from the pGEM-T Easy Vector system (Promega, Madison, WI, USA) was used (Al-Dailami et al., 2022).

Fifth instars were injected with 1 μl containing a total of 2 μg of the two non-overlapping dsLGR1 fragments or 1 μl of 2 µg µl^−1^ dsARG as a control, into the thoracic/abdominal hemocoel at the base of the metathoracic legs using a 10 μl Hamilton microsyringe (Hamilton Company, Reno, NV, USA). Insects were left at room temperature for 1 h to recover and then placed in an incubator at 28°C in the dark.

### MT secretion assay (Ramsay assay)

Whole MTs from dsLGR1- and dsARG-injected insects (2–4 days after dsRNA injection) were excised using fine glass probes and microscissors and transferred into 20 μl droplets of *R. prolixus* physiological saline in a Sylgard-lined Petri dish and overlaid with water-saturated mineral oil. The proximal end of the tubule was pulled out from the saline droplet and wrapped around a minuten pin. The initial bathing saline was replaced with 20 μl saline containing test compounds, which included RhoprCRF/DH, 5HT or RhoprCAPA-2. MTs were nicked gently at the end near to the pin and the secreted droplets from the nicked end were moved using an oil-filled micropipette tip, and then the diameter of the droplets was measured using an eyepiece micrometer. Each sample droplet was collected within 25–30 min. Fluid secretion rates were determined as previously described (Donini et al., 2008). Briefly, the droplet volume was calculated using the equation *V*=(π/6)*d*^3^ where *d* is the diameter of the droplet.

### Feeding assay

Two days after injection with dsRNAs, insects from both treatment groups were weighed and fed on defibrinated rabbit blood for 20 min. Only insects that attempted to feed were used in the feeding assay. Immediately after feeding, insects were weighed to measure the mass of the blood meal consumed and the rate of mass loss was monitored for up to 4 h (240 min) post-feeding (as a proxy for the rate of diuresis).

### Statistical analyses

All graphs were created with GraphPad Prism 9 (GraphPad Software, San Diego, CA, USA). Significance of differences was determined with a one-way ANOVA followed by a Tukey's *post hoc* test to compare changes in transcript levels of LGR1 in segments of MTs in the unfed state, changes in fluorescence intensity as well as fluid secretion rate between different treatment groups. Unpaired Student's *t*-test was used to evaluate the statistical significance in LGR1 knockdown, to compare changes in transcript levels of multiple receptors in different treatment groups, as well as to compare pre- and post-fed body mass between treatment and control groups in the feeding assay. Rate of mass loss after feeding was analyzed by fitting the data with regression lines and comparing the slopes for significant differences using an *F*-test. Differences between treatments were considered significant if *P<*0.05.

## RESULTS

### GBP5-like immunoreactive staining in the MTGM

**Fig. 1.**
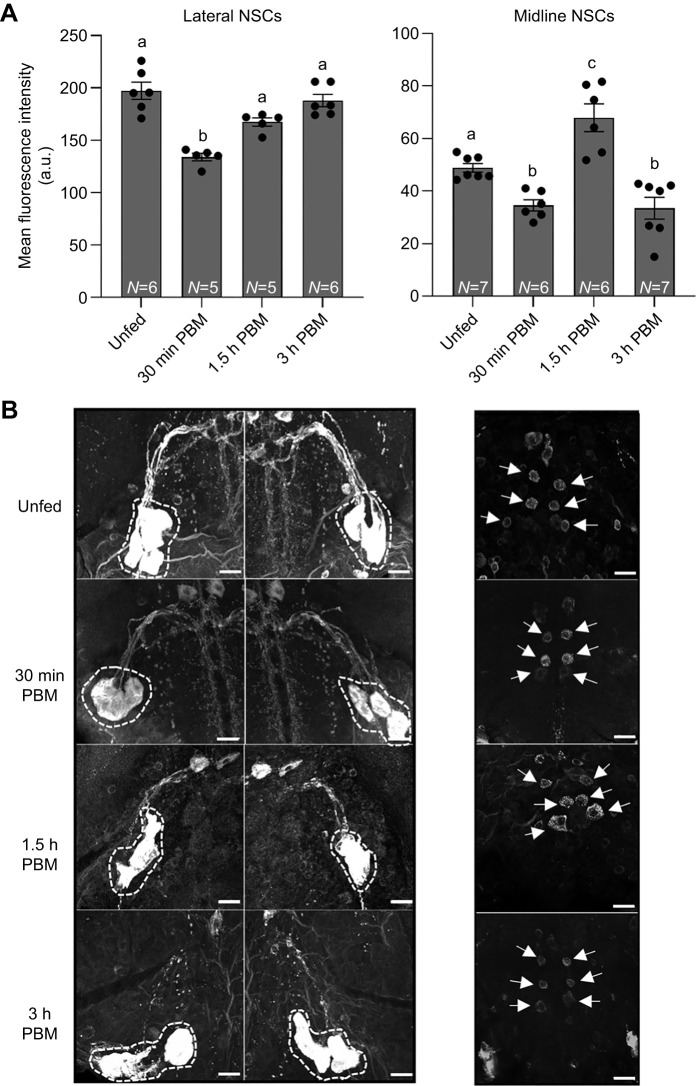
**GPB5-like immunoreactive staining of fifth instar *Rhodnius prolixus* mesothoracic ganglionic mass (MTGM).** (A) Mean fluorescence intensity (a.u., arbitrary units) of stained neurosecretory cells (NSCs) in the lateral and midline of the MTGM in unfed, 30 min post-blood meal (PBM), 1.5 h PBM and 3 h PBM *R. prolixus*. Data are means±s.e.m. of *n*=5–7. Circles indicate data points. Statistical analysis was performed using a one-way ANOVA test with Tukey's multiple comparisons. Significance of *P*<0.05 is denoted using different letters above bars. (B) Representative images showing staining intensity of 5 bilaterally paired NSCs (freeform circled) located dorsally in the MTGM (left) and 3 pairs of NSCs (arrows) in the midline of the ventral side of MTGM (right) under the four conditions. Preparations shown are representative of *n*=5–7. Scale bars: 20 µm.

GBP5-like immunoreactivity has previously been shown to be co-localized with the diuretic hormone RhoprCRF/DH in lateral NSCs of the MTGM and with the anti-diuretic hormone RhoprCAPA-2 in midline NSCs of the MTGM (Al-Dailami et al., 2023, 2024). To examine whether the glycoprotein hormone is released from these NSCs, a time course of immunoreactive staining of fifth instar MTGMs was performed and revealed changes in GPB5-like staining intensity in these NSCs after blood gorging (Fig. 1). Fluorescence intensity of cell bodies, quantified with grayscale values, was significantly reduced in both the midline and lateral NSCs 30 min post-blood meal (PBM) compared with unfed. The intensity of the GPB5-like immunoreactivity in the lateral NSCs then increased, resembling unfed levels by 3 h PBM; however, the intensity of staining in the midline NSCs significantly increased above unfed at 1.5 h PBM, indicating possible restocking of the glycoprotein hormone (Fig. 1). Representative images of GPB5-like immunoreactive staining in the lateral and midline NSCs at each feeding time point are shown in Fig. 1B. It would appear that the glycoprotein is released at feeding.

### *Rhodnius prolixus* LGR1 transcript levels in upper and lower MTs

**Fig. 2.**
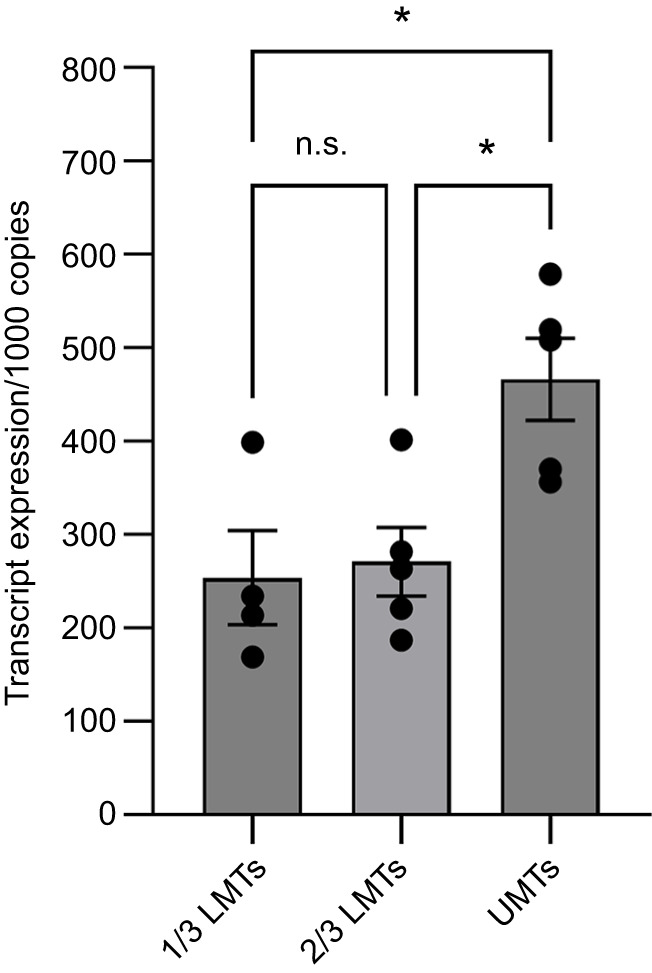
**Transcript levels of LGR1 in different segments of the Malpighian tubules (MTs) of *R. prolixus* fifth instar in the unfed state.** MTs were dissected into upper (distal) MTs (UMT), and lower 1/3 and upper 2/3 of the lower (proximal) MTs (LMTs). Data are means±s.e.m. of *n*=4–5 (each *n* is a pool of MTs from 4 insects). Circles indicate data points. Statistical analysis was performed using a one-way ANOVA test with Tukey's multiple comparisons (*P<0.05; n.s., not significant). Gene expression was quantified using RT-qPCR and the 2^−ΔCT^ method. The *y*-axis represents the expression relative to 1000 copies of the reference genes *rp49* and *β-actin*, obtained via geometric averaging.

A previous study revealed that the LGR1 (RPRC007243) gene is highly expressed in MTs (Orchard et al., 2021) and here we examined in more detail transcript levels in the upper (distal) and lower (proximal) MTs. The complete length of each MT was approximately 53.5±0.4 mm, consisting of the lower MT (21.2±0.4 mm) and the upper MT (32.3±0.3 mm). LGR1 transcript was detected in both the upper MT and the lower MT, including its lower third (Fig. 2), indicating that the glycoprotein hormone signaling pathway may play a role in secretion and reabsorption of ions which occur separately between upper and lower third of the lower MT.

### Effect of *R. prolixus* LGR1 downregulation on basal secretion by MTs

**Fig. 3.**
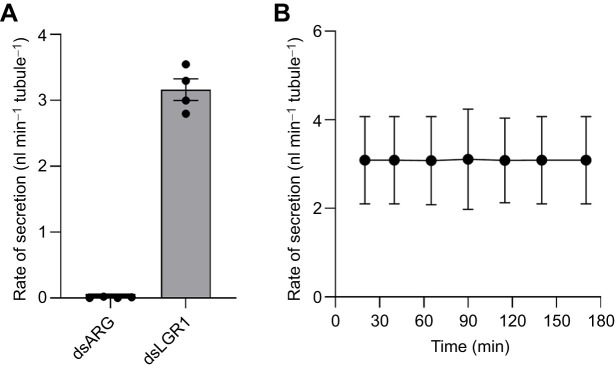
**LGR1 transcript downregulation results in basal fluid secretion by the MTs from unfed *R. prolixus* fifth instars.** (A) Rate of secretion over 20 min from MTs of dsARG- and dsLGR1-injected insects. Data are means±s.e.m. of *n*=4. Circles indicate data points. (B) Rate of secretion from dsLGR1-injected insects showing a constant secretion rate recorded over 180 min. Data are means±s.e.m. of *n*=4.

To determine the effect of GPA2/GPB5 signaling on MTs in unfed insects, LGR1 was downregulated using dsLGR1 injection (Fig. S1) and MT fluid secretion rate examined using the Ramsay assay. MTs from unfed *R. prolixus* have previously been shown to have little to no basal secretion (Te Brugge et al., 2002b), and this was also found to be the case for control insects injected with dsARG (Fig. 3A). However, MTs from *R. prolixus* injected with dsLGR1 did have a basal secretion of 3.2±0.4 nl min^−1^ tubule^−1^ and this secretion rate persisted for up to 3 h (Fig. 3).

### Effect of the anti-diuretic hormone RhoprCAPA-2 on basal secretion following LGR1 downregulation

**Fig. 4.**
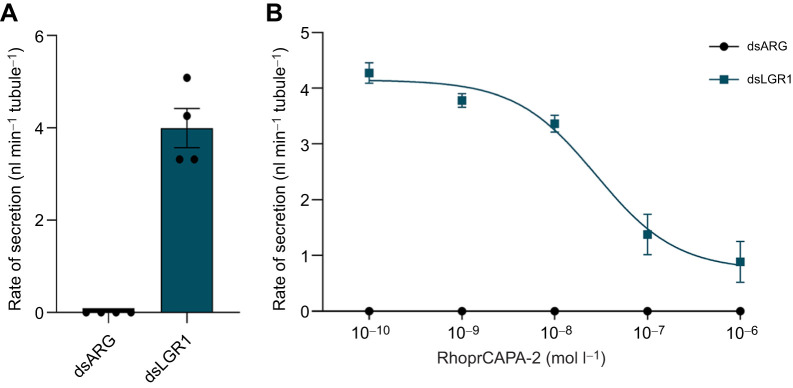
**Fluid secretion rate by MTs from unfed *R. prolixus* fifth instars with downregulated LGR1 transcript in the presence of the anti-diuretic hormone RhoprCAPA-2.** (A) Rate of fluid secretion over 25–30 min from dsARG- and dsLGR1-injected insects. Data are means±s.e.m. of *n*=4. Circles indicate data points. (B) Concentration–response curve of the inhibition of MT fluid secretion rate in the presence of RhoprCAPA-2 of dsARG- and dsLGR1-injected insects. Data are means±s.e.m. of *n*=6.

RhoprCAPA-2 is an antidiuretic hormone in *R. prolixus*, inhibiting fluid secretion by MTs (Paluzzi and Orchard, 2006). We were interested in examining whether RhoprCAPA-2 could inhibit the basal secretion initiated by downregulating LGRI. Fig. 4 shows that RhoprCAPA-2 inhibits the basal fluid secretion rate in dsLGR1-injected insects from 4.0±0.6 nl min^−1^ tubule^−1^ (Fig. 4A) to 0.8±0.4 nl min^−1^ tubule^−1^ (Fig. 4B). This effect was concentration dependent with a threshold at approximately 1 nmol l^−1^ and maximum at approximately 1 µmol l^−1^. IC_50_ was 0.28 nmol l^−1^. Note that CAPA did not affect secretion rate in the dsARG-injected insects.

### Effect of LGR1 downregulation on diuretic hormone-stimulated MT secretion

**Fig. 5.**
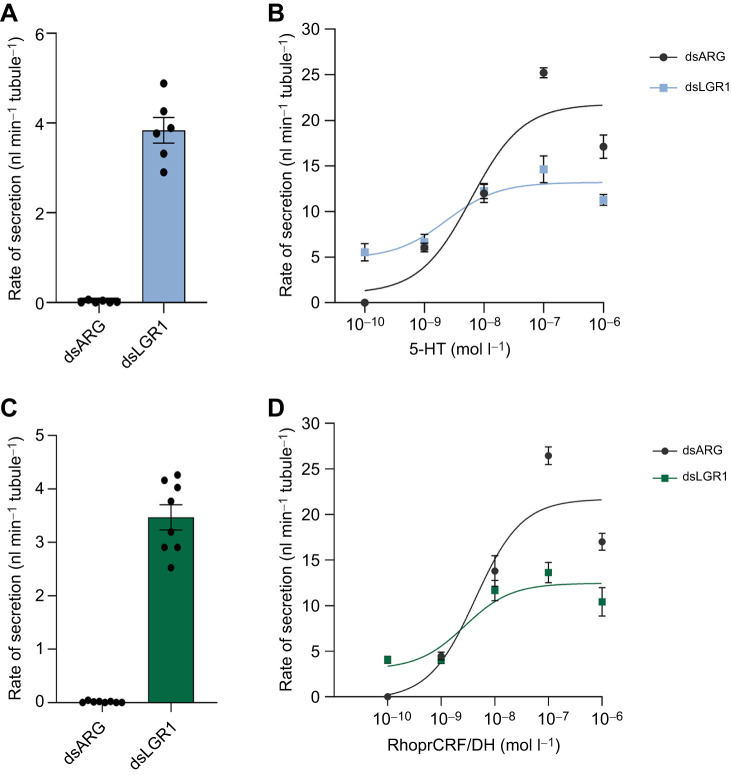
**Fluid secretion rate by MTs from unfed *R.*** ***prolixus*** **fifth instars in the presence of the diuretic hormones 5-HT and RhoprCRF/DH.** (A) Basal rate of fluid secretion over 25–30 min from dsARG- and dsLGR1- injected insects. Data are means±s.e.m. of *n*=6. Circles indicate data points. (B) Concentration–response curves of secretion rate after the addition of 5-HT. Data are means±s.e.m. of *n*=8. (C) Basal rate of fluid secretion over 25–30 min from dsARG- and dsLGR1-injected insects. Data are means±s.e.m. of *n*=8. Circles indicate data points. (D) Concentration–response curves of the fluid secretion rate after the addition of RhoprCRF/DH. Data are means±s.e.m. of *n*=8.

RhoprCRF/DH and 5-HT are true diuretic hormones in *R. prolixus*, and potent stimulators of MT secretion in the Ramsay assay (Te Brugge et al., 2002b, 2011). Interestingly, downregulating LGR1, which resulted in a basal fluid secretion (Fig. 5A,C), reduced the effectiveness of each diuretic hormone at all concentrations tested (Fig. 5B,D), with stimulated secretion rates reaching a maximum of ∼12 nl min^−1^ tubule^−1^ (dsLGR1) compared with ∼21 nl min^−1^ tubule^−1^ observed in the stimulated MTs from control insects (dsARG) for either hormone (Fig. 5).

### Effect of *R. prolixus* LGR1 downregulation on the synergy produced by the two diuretic hormones

**Fig. 6.**
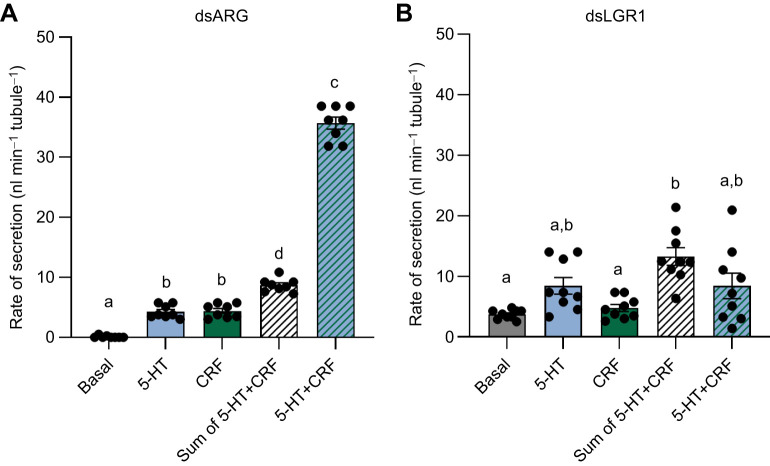
**Fluid secretion rates by MTs from unfed *R. prolixus* fifth instars in the separate/combined presence of the diuretic hormones 5-HT and RhoprCRF/DH.** Rate of secretion over 25–30 min from MTs of dsARG-injected insects (A) and LGR1-injected insects (B) showing the basal rate, and rates after the addition of 10^−9^ mol l^−1^ 5-HT or 10^−9^ mol l^−1^ RhoprCRF/DH (CRF), or a combination of 10^−9^ mol l^−1^ 5-HT and RhoprCRF/DH. Hatched white bar is the arithmetic sum of the secretion rates when the diuretic hormones were applied individually. Data are means±s.e.m. of *n*=8 (dsARG) and *n*=9 (LGR1). Circles indicate data points. Statistical analysis was performed using a one-way ANOVA test with Tukey's multiple comparisons. Significance of *P<*0.05 is denoted using different letters above bars.

To further investigate the involvement of GPA2/GPB5 signaling in MT fluid secretion, we examined the effect of LGR1 downregulation on the known synergistic action of the two diuretic hormones (Paluzzi et al., 2012). In dsARG-injected insects (controls), 10^−9^ mol l^−1^ 5-HT and 10^−9^ mol l^−1^ RhoprCRF/DH each resulted in small increases in secretion by MTs but when applied together they were synergistic, stimulating MT fluid secretion rate to 35.7±1.2 nl min^−1^ tubule^−1^, ∼4-fold greater than the secretion rate expected (Fig. 6) if the effects were additive (8.0±0.5 nl min^−1^). In contrast, downregulating LGR1 eliminated this synergy (Fig. 6).

**Fig. 7.**
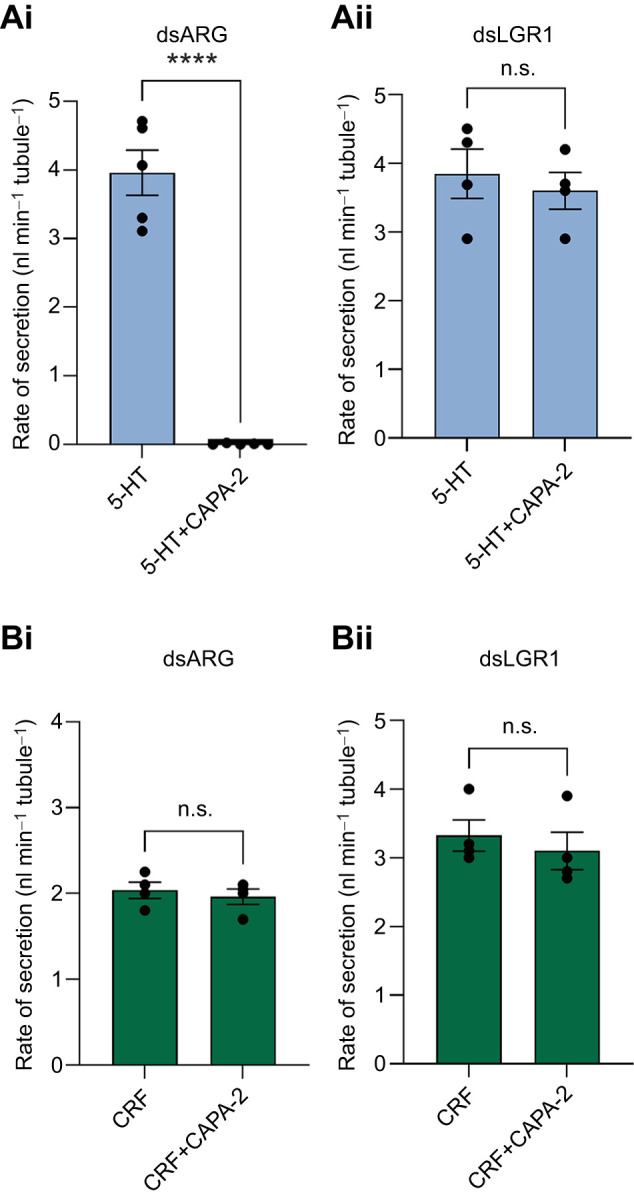
**Fluid secretion rate by MTs from unfed *R. prolixus* fifth instars in the presence of the diuretic hormones 5-HT and RhoprCRF/DH before and after the addition of the anti-diuretic hormone RhoprCAPA-2.** Rate of fluid secretion over 25–30 min before and after the addition of 10^−7^ mol l^−1^ RhoprCAPA-2 (CAPA-2) to MTs of dsARG- or dsLGR-injected fifth instars stimulated with 10^−9^ mol l^−1^ 5-HT (Ai,ii) or 10^−9^ mol l^−1^ RhoprCRF/DH (Bi,ii). Data are means±s.e.m. of *n*=4–5. Circles indicate data points. Statistically significant differences were determined by Student's *t*-test (****P<0.0001; n.s., not significant).

**Fig. 8.**
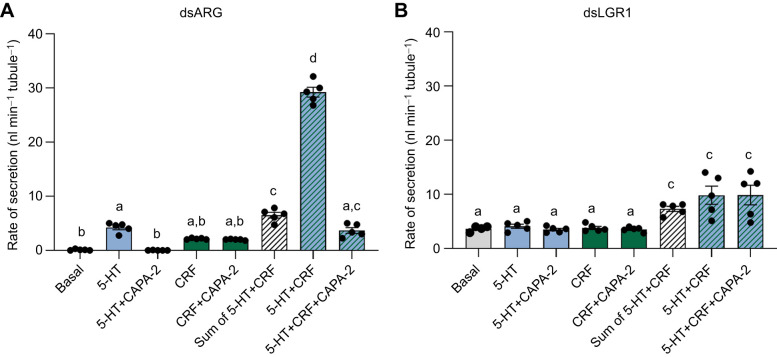
**Fluid secretion rate by MTs from unfed *R. prolixus* fifth instars in the separate/combined presence of the diuretic hormones 5-HT and RhoprCRF/DH before and after the addition of the anti-diuretic hormone RhoprCAPA-2.** Rate of secretion over 25–30 min from (A) dsARG- and (B) dsLGR1-injected fifth instars after the addition of 10^−9^ mol l^−1^ 5-HT and 10^−9^ mol l^−1^ RhoprCRF/DH individually or combined, and in the presence of 10^−7^ mol l^−1^ RhoprCAPA-2. Hatched white bar is the arithmetic sum of the secretion rates calculated when diuretic hormones were applied individually. Data are means±s.e.m. of *n*=5. Circles indicate data points. Significance of *P<*0.05 is denoted using different letters above bars.

In light of the data above, we tested the influence of dsLGR1 on the ability of the anti-diuretic peptide RhoprCAPA-2 to inhibit the MT fluid secretion stimulated by the diuretic hormones 5-HT and RhoprCRF/DH individually (Fig. 7) or combined (Fig. 8). Similar to previous results (Paluzzi et al., 2012), 10^−7^ mol l^−1^ RhoprCAPA-2 inhibited fluid secretion stimulated by 10^−9^ mol l^−1^ 5-HT in dsRNA controls (Figs 7Ai and 8A) but did not inhibit secretion stimulated by 10^−9^ mol l^−1^ RhoprCRF/DH in dsARG- or in dsLGR1-injected insects (Fig. 7Bi,ii). Interestingly, no inhibition by RhoprCAPA-2 of 5-HT-stimulated secretion of MTs was observed in dsLGR1-injected insects (Figs 7Aii and 8B). RhoprCAPA-2 had no effect on the secretion rate in MTs seen with the combined addition of 5-HT and RhoprCRF/DH in dsLGR1-injected insects (Fig. 8B).

### Effect of *R. prolixus* LGR1 downregulation on 5-HTR2b, CRFR2 and CAPAR transcript levels in MTs of unfed and fed insects

**Fig. 9.**
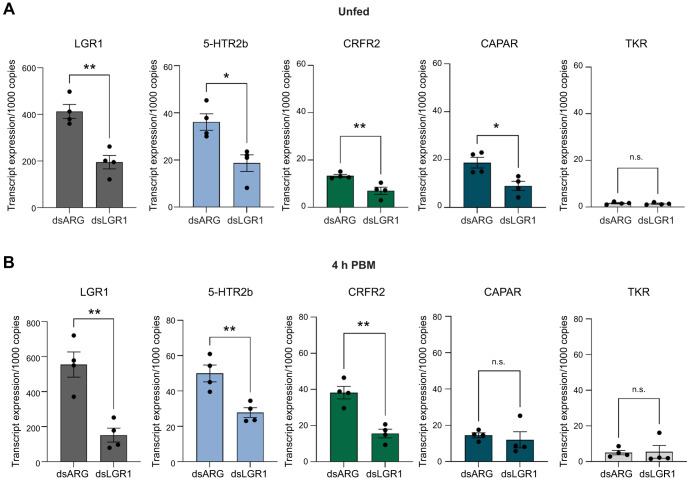
**Transcript levels of LGR1, 5-HT receptor (5-HTR2b), RhoprCRF/DH receptor (CRFR2), RhoprCAPA-2 receptor (CAPAR) and tachykinin receptor (TKR) in MTs of dsARG- versus dsLGR1-injected *R. prolixus* fifth instars, unfed and 4 h PBM.** (A) Transcript levels of the receptors in dsARG- and dsLGR1-injected insects in the unfed state. (B) Transcript levels of the receptors in dsARG- and dsLGR1-injected insects at 4 h PBM. Data are means±s.e.m. of *n*=4 (each *n* is a pool of MTs from one insect). Circles indicate data points. Statistically significant differences were determined by Student's *t*-test to compare changes in transcript levels of receptors in treatment and control groups (**P<0.01; *P<0.05; n.s., not significant). Gene expression was quantified using RT-qPCR and the 2^−ΔCT^ method. The *y*-axis represents the expression relative to 1000 copies of reference genes *rp49* and *β-actin*, obtained via geometric averaging.

We quantified the transcript levels of the anti-diuretic hormone receptor RhoprCAPA-R (CAPAR; RPRC000516), and the diuretic hormone receptors RhoprCRF/DH-R2 (CRFR2; RPRC000578) and Rhopr5HT-R2b (5-HTR2b; RPRC000473) in dsARG- and dsLGR1-injected unfed insects and at 4 h PBM (Fig. 9). As expected, downregulation of LGR1 in MTs of unfed insects resulted in a significant reduction of 53% of LGR1 transcript levels (Fig. 9A). Interestingly, a significant reduction was also observed in 5-HTR2b, CRFR2 and CAPAR transcript levels in MTs of unfed insects injected with dsLGR1 compared with the dsARG controls (Fig. 9A). As a control, we also quantified the tachykinin receptor (TKR; RPRC008022) transcript levels as there is no evidence of tachykinin altering secretion rate. No significant changes in TKR transcript levels were observed in MTs from unfed insects treated with dsLGR1 (Fig. 9A), indicating that not all GPCRs in MTs are downregulated by dsLGR1 treatment.

At 4 h PBM, transcript levels of LGR1 remained significantly reduced by 71% and levels of 5HTR2b and CRFR2 also remained significantly reduced in insects injected with dsLGR1 compared with the control (Fig. 9B). CAPAR transcript levels were no longer significantly reduced (Fig. 9B). Similar to what was seen in unfed insects, TKR transcript levels were not significantly reduced in MTs 4 h PBM in insects treated with dsARG or dsLGR1 (Fig. 9B).

### Effect of *R. prolixus* LGR1 downregulation on feeding and mass loss (diuresis)

**Fig. 10.**
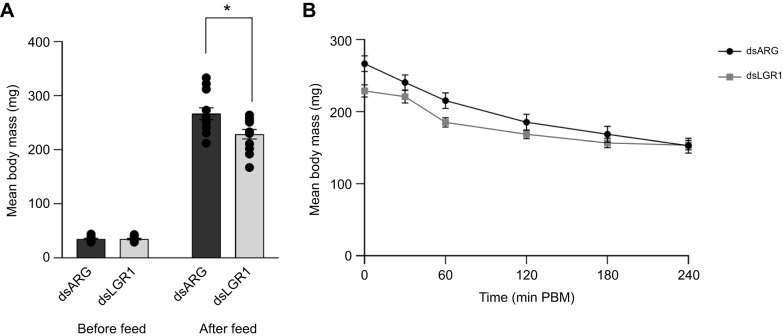
**The effect of LGR1 knockdown on *R. prolixus* feeding and diuresis (as monitored by mass loss) using dsRNA. (**A) Blood meal size consumed by dsARG- and dsLGR1-injected insects. Data are means±s.e.m. (*n*=12 for each treatment group). Circles indicate data points. Statistically significant differences were determined using Student's *t*-test to compare post-feeding body mass between treatment and control groups (*P<0.05). (B) Mean body mass of dsARG- and dsLGR1-injected insects over the first 240 min PBM. Data are means±s.e.m. (*n*=12 for each treatment group). The significance of differences between linear regression of slopes of dsLGR1-injected and dsARG-injected groups was determined using *F*-test (*P=*0.033).

dsLGR1-injected insects took a significantly smaller blood meal compared with dsARG-injected insects, with a mean body mass of 228.7±8.3 mg in dsLGR1-injected insects compared with 266.7±10.3 mg in dsARG-injected insects (*P=*0.0008) (Fig. 10). We monitored the mean body mass of both groups PBM (as a proxy for diuresis) and found that in dsLGR1-injected insects, body mass did not decrease over the first 30 min PBM compared with the sharp decrease in mean body mass of the control insects. The slope of the linear regression line was measured and found to be statistically significant (*F*-test, *P*=0.033) between the two groups, suggesting dsLGR1 influences post-prandial diuresis (Fig. 10).

## DISCUSSION

Glycoprotein hormones are highly conserved neuroendocrine factors (Rocco et al., 2019; Hausken and Levavi-Sivan, 2019; Kenis et al., 2023; Istiban et al., 2024). In vertebrates, five heterodimeric glycoprotein hormones have been identified, namely thyroid-stimulating hormone, follicle-stimulating hormone, luteinizing hormone, chorionic gonadotropin and thyrostimulin (Cahoreau et al., 2015). The first four are restricted to vertebrates, but orthologs of thyrostimulin's subunits GPA2 and GPB5 are also found in invertebrates (Roch and Sherwood, 2014; Istiban et al., 2024). GPA2 and GPB5 are considered the ancestral glycoprotein hormone subunits that evolved in a common ancestor of bilaterian animals (Cahoreau et al., 2015; Roch and Sherwood, 2014). Thyrostimulin's ancestral origin is corroborated by the broad conservation of GPA2/GPB5 receptors, named LGR1 in arthropods and FSHR1 in nematodes. In vertebrates and arthropods, thyrostimulin (GPA2/GPB5)-like signaling has pleiotropic roles in reproduction, development, growth, immunity and ion homeostasis (Paluzzi et al., 2014; Vandersmissen et al., 2014; Rocco et al., 2019; Wahl et al., 2022; Al-Dailami et al., 2022). In *Caenorhabditis elegans*, FSHR-1 is involved in immune and stress responses, gut functioning, growth and reproduction (Istiban et al., 2024).

GPB5-like immunoreactive neurons are present throughout the CNS of unfed fifth instars (Al-Dailami et al., 2022), and in particular these neurons include the lateral NSCs that contain and release the diuretic hormone RhoprCRF/DH (Te Brugge and Orchard, 2002a) and the midline NSCs that contain and release the antidiuretic hormone RhoprCAPA-2 (Paluzzi and Orchard, 2006). Previous studies have shown staining of the CRF-like NSC is diminished immediately following a blood meal (Mollayeva et al., 2018; Te Brugge and Orchard, 2002b) and staining of the CAPA-like NSCs decreases at 3 h post-feeding – a time that coincides with cessation of diuresis (see Paluzzi and Orchard, 2006). Co-localization of GPB5-like immunoreactivity is found in each of these NSC groups and also exists in the neurohemal release sites on abdominal nerves of the respective diuretic or anti-diuretic hormone. Here, we provide supporting evidence that the glycoprotein is released at feeding, with the staining intensity of the neuronal cell bodies decreasing after feeding and then increasing, likely as a result of restocking. A link between GPA2/GPB5 release from lateral and midline NSCs in the MTGM and feeding is also evident in our feeding assay, whereby downregulation of LGR1, using dsLGR1 injection, decreases blood meal intake and disturbs the timing of post-prandial rapid diuresis.

A transcriptomic analysis of *R. prolixus* MTs indicates LGR1 to be the most abundant GPCR transcript (Orchard et al., 2021), further suggesting a role for the glycoprotein signaling pathway in MT physiology. Here, we show that the transcript is present in the lower MT (including its lower third) and in the upper MT, suggesting the glycoprotein hormone signaling pathway may play a role in the secretion and reabsorption of ions which occurs separately between upper and lower MTs, respectively. Indeed, downregulation of LGR1 in unfed insects results in a basal fluid secretion rate by MTs which is not normally seen in *R. prolixus* MTs of unfed insects and was not seen in control insects here. RhoprCAPA-2 inhibits the basal secretion rate of dsLGR1 MTs. This induced basal secretion may explain an earlier result whereby unfed insects injected with dsLGR1 had a loss of body mass and a significant increase in mortality rate over time, suggesting these insects might be suffering from loss of water and thereby dehydration stress (Al-Dailami et al., 2022). Thus, in unfed *R. prolixus*, the glycoprotein signaling pathway might be used to ensure the MTs are in a minimal state of secretion, an idea supported by the increasing levels of the LGR1 transcript in the MTs as the unfed state is extended (Al-Dailami et al., 2022). Similarly, in *Drosophila melanogaster*, silencing of LGR1 results in a notable decrease in water content and survival rate under desiccating conditions (Vandersmissen et al., 2014). Paluzzi et al. (2014) also proposed that the GPA2/GPB5 hormonal system regulates ion and water balance by influencing epithelial transport in the alimentary canal of *Aedes aegypti*. It is interesting that in *A. aegypti* and *D. melanogaster* it has been suggested that LGR1 is constitutively active; that is, having actions in the absence of its ligand, GPA2/GPB5 (Nishi et al., 2000; Sudo et al., 2005; Rocco and Paluzzi, 2020). Thus, knocking down LGR1 in unfed *R. prolixus* would remove this constitutive activity, which might eliminate an inhibitory control over secretion, thereby producing the basal secretion observed.

Fluid secretion by MTs is stimulated by the diuretic hormones Rhopr-CRF/DH and 5-HT (individually and synergistically when combined), with the anti-diuretic hormone RhoprCAPA-2 inhibiting 5-HT-stimulated secretion and the synergy produced by the two diuretic hormones (Paluzzi and Orchard, 2006; Paluzzi et al., 2012; Orchard et al., 2021). Interestingly, knocking down expression of the gene for LGR1 eliminated the synergy that is normally seen when the diuretic hormones are applied together, and reduced the effectiveness of the diuretic hormones applied individually. Also, RhoprCAPA-2 was no longer effective at inhibiting 5-HT-stimulated secretion of MTs in dsLGR1-injected insects. To further examine the influence of LGR1 downregulation on the actions of diuretic and antidiuretic hormones, we quantified transcript levels of their receptors in dsLGR1-injected unfed and fed insects. A notable reduction in Rhopr5HT-R2b, RhoprCRF/DH-R2 and RhoprCAPA-R transcript levels in MTs of unfed and fed insects (except for RhoprCAPA-R in the fed insect) was found using dsLGR1 compared with the control, suggesting a possible mechanism for the lowering of biological activity of the diuretic and antidiuretic hormones. Thus, glycoprotein hormone signaling appears to play a critical role in tightly regulating the complex interactions between the diuretic hormones RhoprCRF/DH and 5-HT and the anti-diuretic hormone RhoprCAPA-2.

Previous analyses of MTs have also revealed the presence of transcripts that are distinct from those likely to control diuresis (Esquivel et al., 2016; Dow and Davies, 2006; Dow et al., 2018; Xu et al., 2022). Thus, microarrays, transcriptomes and proteomics illustrate additional major roles that MTs play in insect immunity, detoxification, pesticide resistance and tolerance to overall stress. This is particularly relevant to *R. prolixus* as blood gorging creates several challenges, including osmotic balance, xenobiotics in the blood or toxins produced by metabolism of blood (see Orchard et al., 2023). These all need to be neutralized and excreted. It is possible, given the role of glycoprotein hormones in other invertebrates in immunity (Istiban et al., 2024), that the signaling pathway in *R. prolixus* MTs may coordinate aspects of diuresis as well as their likely roles in immunity and detoxification. This will be an important direction for future studies.

It is worth reiterating that the GPA2/GPB5 signaling system is evolutionarily ancient and is present in all bilaterians (Santos et al., 2011; Hauser et al., 1998; Vibede et al., 1998), suggesting its pivotal role in maintaining an overall physiological homeostasis in diverse species. In *R. prolixus* fifth instars, this manifests itself by the requirement of the MTs to express LGR1 in order to survive the unfed condition, and to coordinate the actions of the diuretic and antidiuretic hormones at the appropriate time, thereby ensuring successful post-prandial diuresis. Thus, the co-localization of the glycoprotein hormone with the diuretic and antidiuretic hormones and their co-release suggests LGR1 activity changes over time to influence the activity of the diuretic and antidiuretic hormones for fine tuning of diuresis.

## Supplementary Material

10.1242/jexbio.249357_sup1 Supplementary information
