# Supplementary material for: The glycoprotein hormone receptor (LGR1) influences Malpighian tubule secretion rate in Rhodnius prolixus
Source: J Exp Biol. 2024 Dec 10;227(24):jeb249357. doi: 10.1242/jeb.249357 (PMC11655026; doi:10.1242/jeb.249357)
Supplement: Supplementary information [file jexbio-227-249357-s1.pdf]

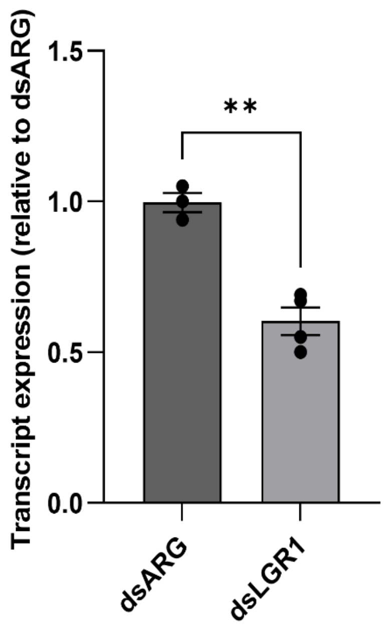

Fig. S1. Verification of dsRNA efficiency by real time qPCR using $2^{-\Delta\Delta Ct}$ method. Transcript levels of *Rhodnius prolixus* glycoprotein hormone receptor, LGR1, in the Malpighian tubules (MTs) of unfed insects at 2 days post dsLGR1 injection. LGR1 is reduced by 43% compared to the controls (dsARG- injected insects). Symbols are mean ± SEM of n = 3-4. Circles indicate data points. Statistically significant difference was determined by Student's t-test. ***P* < 0.01. The y axis represents transcript expression of LGR1 in MTs from dsARG- and dsLGR1- injected insects calculated as fold change relative to dsARG, obtained via geometric averaging of the reference genes Rp49 and β-actin.

Table S1. Gene-specific primers used for qPCR and dsRNA experiments.

Oligo Name	Oligo Sequence 5'- 3'
LGR1 F	CCGCCAGATAATGGACCTTGT
LGR1 R	TTCGTGAATCATGTGTTCTTCA
LGR1 (2) F	ATTATAATGGCGGCCCTACC
LGR1 (2) R	TGGGCATATCGCGTACATAA
CRFR2 F	ACTGCTCTTGGTTTGGCAGT
CRFR2 R	GCCGGGTCTGTTTTAACGTA
5-HTR2b F	CTTAGACGCGACGATCACAA
5-HTR2b R	TTGTGCTGCTTCTTCCATTG
TKR F	TCAACAACTACCTGGCAAA
TKR R	ACTGGGTTTGTGTGTAATGG
Rp49 F	ACCAATGGAAGTAACCGCCT
Rp49 R	AGGACACACCATGCGCTATC
Actin F	AGAGAAAAGATGACGCAGATAATGT
Actin R	ATATCCCTAACAATTTCACGTTCG
CAPAR F	TCATTTCAGGACTATTTGGTAATTTAGC
CAPAR R	GAGACGTAGGATGACATTTCTGAG
ARG F	ATGAGTATTCAACATTTCCGTGTC
ARG R	AATAGTTTGCGCAACGTTG
LGR1_F_T7	**TAATACGACTCACTATAGGGAGA** CCGCCAGATAATGGACCTTGT
LGR1_R_T7	**TAATACGACTCACTATAGGGAGA**TTCGTGAATCATGTGTTCTTCA
LGR1(2)_F_T7	**TAATACGACTCACTATAGGGAGA** ATTATAATGGCGGCCCTACC
LGR1(2)_R_T7	**TAATACGACTCACTATAGGGAGA** TGGGCATATCGCGTACATAA
ARG F_T7	**TAATACGACTCACTATAGGGAGA**ATGAGTATTCAACATTTCCGTGTC
ARG R_T7	**TAATACGACTCACTATAGGGAGA**AATAGTTTGCGCAACGTTG

* **TAATACGACTCACTATAGGGAGA** = T7 RNA polymerase promoter region

Journal of Experimental Biology • Supplementary information
